# Factors associated with recognition of the signs of dating violence by Japanese junior high school students

**DOI:** 10.1007/s12199-015-0491-1

**Published:** 2015-09-26

**Authors:** Miyuki Nagamatsu, Yukiko Hamada, Kenichi Hara

**Affiliations:** Department of Maternal and Child Nursing, Faculty of Medicine, Saga University, 5-1-1, Nabeshima, Saga, 849-8501 Japan; Department of Maternal Nursing, Faculty of Health Sciences, Junshin Gakuen University, Fukuoka, Japan; Saga Prefectural Center for General Countermeasure, Against Domestic Violence, Saga, Japan

**Keywords:** Adolescents, Dating violence, Recognition, Knowledge, Relationship

## Abstract

**Objectives:**

This study investigated factors associated with the ability of Japanese junior high school students to recognize the signs of dating violence.

**Methods:**

During a period of 20 months (from June 2011 to January 2013), a survey was distributed to 3340 students aged 13–15 years in the second and third grades at 18 junior high schools in a Japanese prefecture. The survey examined gender, recognition of the signs of dating violence, knowledge of dating violence, self-esteem, attitudes toward sexual activity, attitudes toward an equal dating relationship, and relationships with school teachers. Multiple linear regression analyses were performed to identify predictors of the ability of boys and girls respondents to recognize the signs of physical and psychological dating violence. Binary  multiple logistic regression analysis was also performed to identify predictors of the ability of boys and girls respondents to recognize the sign of sexual dating violence. The Ethics Committee of Saga University Medical School approved the study protocol.

**Results:**

A total of 3050 (91.3 %) students participated in this study (1547 boys and 1503 girls). Gender differences were noted with regard to the scores for some of the variables measured. The results indicated that boys who had more knowledge of dating violence, who focused on an equal dating relationship, and had a positive relationship with their teachers showed a greater ability to recognize the signs of dating violence. In addition, boys with a conservative attitude toward sexual activity showed a greater ability to recognize the signs of physical and sexual violence. Furthermore, girls with more knowledge of dating violence had a conservative attitude toward sexual activity, and girls who focused on an equal dating relationship showed greater ability to recognize the signs of dating violence.

**Conclusions:**

These findings suggest that education programs to prevent dating violence should promote understanding about dating violence with consideration of gender differences and should foster better relations between students and teachers, as well as promoting the establishment of an equal dating relationship between boys and girls.

## Introduction

Dating violence has become one of today’s most serious and fastest increasing social and health concerns. The prevalence of dating violence among adolescents has been reported, with one in five female high school students reporting being physically and/or sexually assaulted by a date [1]. Given that younger age is a consistent predictor for experiencing dating violence and that physical and sexual violence by a dating partner is prevalent among high school girls, adolescence is a particularly vulnerable time for dating violence. In Japan, for instance, 13.6 % female and 4.3 % male adult participants reported being victims of dating violence [2]. In Matsuda’s study [3], it is reported that 17.8 % male and 16.7 % female Japanese college students admitted that they perpetrated violence against their dating partners. Furthermore, Nakata found that 14 % of college females and 10 % of high school females who participated in the study experienced dating violence, with 30 % of participants who reported of experiencing unwanted sexual activities [4]. Silverman et al. concluded that the experience of dating violence is significantly associated with adolescents’ health risk behaviors, such as substance use, unhealthy weight control, and suicidality [1]. Given the fact that there has been a drastic increase in early dating, and thus early sexual engagement among Japanese adolescents [5] as well as the detrimental consequences of dating violence on adolescents’ well-being, early prevention of dating violence is a major and urgent challenge for today’s Japanese society.

One of the difficulties in investigating attitudes toward dating violence is how it is defined. For instance, some researchers include psychological and emotional violent behavior that occur in dating relationships in their definition of dating violence [6], while others use a more restrictive definition that includes only physical violence [7]. In the recent literature, more contemporary definitions have begun to include physical, sexual, and psychological forms of violence [8]. As such, for the purpose of this study, we included all three areas of violence given that the consequences of all three forms of violence are significant detrimental factors on adolescents’ development.

In recent years, the WHO has recommended the enhancement of measures to prevent adolescents from becoming a victim of violence/sexual violence by an intimate partner or becoming a perpetrator of such violence after their first sexual experience [9]. Based on the results of a 2011 survey performed by the Japanese Association for Sex Education [10], the percentage of Japanese teenage boys and girls with sexual experience shows a marked increase from 3.7 to 4.7 %, respectively, among junior high school students to 14.6 and 22.5 % among high school students. It is thought that enhancing the recognition of the signs of dating violence by junior high school students before their first sexual experience is effective education for preventing violence. However, sex education covering dating violence is not mandatory in Japanese junior high schools, so only a small number of junior high school students receive such education and there have only been a few studies about dating violence among junior high school students [11]. As Theriot [12] stated, dating violence among adolescents is a serious form of school violence. It is important for junior high school students to be able to easily recognize the warning signs of such violence and seek proper help from their formal and informal support systems. Thus, it is crucial for school social workers and educators to create and assess strategies for tackling this problem at school.

We hoped to find predictors of the ability to recognize the signs of dating violence, so that effective prevention programs could be provided to Japanese junior high school students in order to reduce dating violence. In Japan, there have not been any investigations of the factors predicting dating violence among junior high school students. Therefore, the present study was performed to identify factors associated with the ability of Japanese junior high school students to recognize the signs of dating violence.

## Materials and methods

### Participants

The junior high schools which participated in this study were located in the Saga Prefecture (Japan), which is a rural district with an area of 2439 km^2^ and a population of approximately 866,000. Twenty-five schools were randomly selected from among 99 junior high schools and data were collected from 18 schools that agreed to participate in this study. At their junior high schools, no information about dating violence had been provided in sex education classes. During a period of 20 months (from June 2011 to January 2013), a questionnaire survey was distributed to 3340 students aged from 13 to 15 years. Information on prevention of dating violence was provided to their junior high schools after the survey was conducted.

### Instruments

#### Attribute

First, we asked about the gender of the students.

#### Recognition of the signs of dating violence (RSDV)

The RSDV was originally developed by the Government of Japan Cabinet Office to assess students’ ability to recognize the signs of dating violence compiling data from 19,398 Japanese high school students from 80 different schools. Recently, the RSDV has been modified and used by Hara et al. [11] to be appropriate for junior high school students. In this study, participants were asked with a question of more comprehensible expression: do you think that the following behaviors are violence when a romantic partner engages in these behaviors toward the other? In the RSDV, participants are presented with ten behaviors. The RSDV assesses respondents’ ability to recognize the signs of dating violence on three subscales: (1) physical violence, (2) psychological violence, and (3) sexual violence within intimate relationships. Four items were used to assess attitudes on physical violence: (a) injuring by hitting, (b) punching or kicking without inflicting injury, (c) thrusting away or throwing something, and (d) breaking things or pretending to hit. Five items were used to assess attitudes on psychological violence: (e) yelling loudly, (f) ridiculing or mocking to hurt, (g) completely ignoring by not responding to any request, (h) taking freedom by monitoring or not allowing to go out, and (i) becoming irate when the partner talks to or acts friendly with a person of the opposite sex. One item was used to assess attitudes on sexual violence: (j) forcing the partner to not reject sexual advances, such as sexual intercourse or kissing. Participants are instructed to indicate their beliefs about each behavior by indicating 0 = *No* (It is not violence) or 1 = *Yes* (It is violence). The ten items were summed, and the total scores ranged from 0 to 10, with a higher score representing greater ability to recognize dating violence. The Cronbach’s alpha was 0.79 in this study.

#### Knowledge of dating violence (KDV)

The KDV was originally created by Yamaguchi [13] to assess Japanese high school students’ degree of understanding of dating violence. The original scale consisted of items that focused on assessing female high school students’ knowledge of dating violence. Therefore, in the present study, five items were selected after discussion with teachers and intimate partner violence experts because these items were applicable for junior high school students and evaluating the understanding of both male and female students. The selected items were modified to be easily understood by junior high school students by changing words and simplifying expressions. The questions are as follows: (1) Dating violence occurs only in adult couples, (2) Dating violence is not a problem for junior high school or high school students, (3) There are few adolescents who have experienced violence by their dates, (4) If dating violence occurs, it must be only a one-time incident, (5) Dating violence occurs only when a couple hates each other and decides to break up. Students were asked to respond to each of the five statements by indicating 0 = *Yes* (I do think so) or 1 = *No* (I do not think so). All five items were summed, and the total scores ranged from 0 to 5, with a higher score representing greater understanding of dating violence. The Cronbach’s alpha was 0.71 in this study.

#### Self-esteem

The General Self-esteem Scale was used to assess respondents’ self-esteem and consisted of 10 items by Rosenberg [14]. It was translated into Japanese by Hoshino, and its validity has been demonstrated with Japanese participants [15]. This scale has been commonly used for school education to investigate high-risk behavior, such as sexual behavior and relevant factors, in Japanese junior high school and high school students [16]. The response options ranged from 1 (*strongly disagree*) to 4 (*strongly agree*). All 10 items were summed, and the total score ranged from 10 to 40 points with higher scores indicating higher self-esteem. The Cronbach’s alpha was 0.78 in this study.

#### Conservative attitude toward sexual activity (CATSA)

The CATSA scale was developed by Nagamatsu et al. [17] and consists of three items that assess junior high school students’ attitudes of respondents toward engaging in sexual activity. These items are (1) “Do you accept the idea of having sex while in junior high school?” (2) “Do you accept the idea of having sex before graduation from high school?” and (3) “Are you confident to reject sexual advances if you are asked?” Respondents were asked to rate the first two questions with a Likert scale by indicating 1 = *Acceptable*, 2 = *Somewhat acceptable*, 3 = *Somewhat unacceptable*, and 4 = *Unacceptable.* The last question was rated on a Likert scale by indicating 1 = *Not confident*, 2 = *Somewhat not confident*, 3 = *Somewhat confident*, and 4 = *Confident*. The scores for all three items were summed to obtain a total score ranging from 3 to 12 points, with higher scores representing more conservative attitudes. The Cronbach’s alpha was 0.78 in the present study.

#### Attitude toward equal dating relationship (ATEDR)

The ATEDR scale was developed by Nagamatsu et al. [18] and consists of three items that assess junior high school students’ attitude of respondents toward a healthy dating relationship. These items are as follows: (1) How important do you think an equal dating relationship between dating partners is? (2) How important do you think that caring for a partner is? and (3) How important do you think that caring for oneself is? The response options used a Likert scale and ranged from 1 = *Not important*, 2 = *Not so important*, 3 = *Slightly important*, and 4 = *Important*. All three items were summed, and the total score ranged from 3 to 12 points, with higher scores indicating greater importance given to an equal partnership in a dating relationship. The Cronbach’s alpha for this scale was 0.78.

#### Positive relationship with school teachers (PRWST)

The PRWST scale was developed by Nagamatsu et al. [18] and consists of three items that assess communication with school teachers. (a) “Did you talk to any teacher at your school in the past 3 months?”; (b) “Did you talk to any teacher at your school about life in the past 3 months?”; (c) “Did any teacher at your school listen to your opinion in the past 3 months?” Students responded on a 4-point Likert-type scale ranging from 1 = *never* to 4 = *often* to the following questions. All three items were summed, and the total score ranged from 3 to 12 points, with higher scores representing high levels of relationship by more frequency of communication with school teachers. The Cronbach’s alpha was 0.72 in the present study.

### Procedure and ethical considerations

The Ethics Committee of Saga University Medical School approved the study protocol. The survey package was only distributed to each school with the principal’s approval. This package included (1) the questionnaires; (2) an explanatory document for school teachers that gave instructions about how to distribute and retrieve the questionnaires; and (3) consent forms and explanatory documents for students that addressed the objectives of the study, emphasized confidentiality, acknowledged freedom to refuse to participate, and provided information on contacting the researchers. To ensure confidentiality, each teacher left the classroom and remained in the hallway when students were responding to the survey and only retrieved the survey forms after the students had sealed these documents in envelopes. Students could refuse to participate in the survey if they did not want to perform it. Students were told that they did not have to complete the questionnaire unless they wished to, and students who did not give assent were not forced to complete or submit the questionnaire. Students were deemed to have provided consent by placing a questionnaire into the collection bag.

### Analysis

A series of *t* tests were performed to examine gender differences of the measured variables. In addition, multiple linear regression analyses were performed to identify predictors of the ability of boys and girls respondents to recognize the signs of physical and psychological dating violence: the KDV scale, self-esteem, the CATSA scale, the ATEDR scale, and the PRWST scale. Because recognition of the sign of sexual dating violence was measured by one item using a binary scale,  binary multiple logistic regression analysis was also performed to identify predictors of the ability of boys and girls respondents to recognize the sign of sexual dating violence. The Statistical Package for the Social Sciences (SPSS 21.0) was employed for all analyses. Significance of differences was accepted at *p* < 0.05.

## Results

### Gender differences of variables

This study participated 3050 of 3340 students (91.3 %) including 1547 boys and 1503 girls. Table 1 shows means, standard deviations, and gender differences of all measures.

**Table 1:** Means, standard deviations, and gender differences of all measures

Measured variables (minimum–maximum)	Boys	Girls	Boys versus girls	
	(*n* = 1547)	(*n* = 1503)	(*n* = 3050)	
	*M* (SD)	*M* (SD)	*t*	*p* value
Knowledge of dating violence total score (0–5)	3.3 (1.6)	3.9 (1.3)	−11.8	<0.001
Self-esteem total score (10–40)	25.9 (4.9)	24.0 (5.3)	9.8	<0.001
Conservative attitudes toward sexual activities total score (3–12)	9.1 (2.6)	9.1 (2.4)	0.1	0.992
Attitudes toward equal dating relationship total score (3–12)	10.3 (1.8)	10.6 (1.7)	−5.4	<0.001
Positive relationship with school teacher total score (3–12)	8.1 (1.8)	8.2 (1.8)	−2.1	0.033
Recognition of dating violence signs total score (0–10)	7.5 (2.5)	7.4 (2.3)	1.3	0.199
Physical violence (0–4)	3.4 (0.9)	3.4 (0.8)	1.1	0.263
Psychological violence (0–5)	3.4 (1.6)	3.3 (1.5)	1.3	0.194
Sexual violence (0–1)	0.8 (0.4)	0.8 (0.4)	−0.6	0.524

Coding: boy = 1, girl = 0

Compared with the boys, the girls showed significantly higher scores on the KDV scale (*p* < 0.001), as well as having significantly higher scores for the ATEDR scale (*p* < 0.001) and the PRWST scale (*p* = 0.033). In contrast, boys had a significantly higher level of self-esteem than girls (*p* < 0.001). However, there were no gender differences with regard to the scores of the students on the RSDV and CATSA scales.

### Predictors of recognizing of the signs of physical, psychological, and sexual dating violence

Table 2 shows the results of multiple linear regression analysis and binary multiple logistic regression analysis for boys and Table 3 summarizes the findings for girls.

**Table 2:** Multiple regression and binary logistic regression analyses of the effects of selected factors on the recognition of dating violence sings among boys

Boys (*n* = 1547)	Physical violence				Psychological violence				Sexual violence			
	*β*	SE	*t*	*p* value	*β*	SE	*t*	*p* value	Exp (B)	SE	95 % CI	*p* value
Knowledge of dating violence	0.115	0.010	4.257	<0.001	0.164	0.019	5.994	<0.001	1.075	0.029	1.016–1.137	0.012
Self-esteem	0.027	0.005	0.970	0.332	0.003	0.010	0.118	0.906	0.989	0.015	0.960–1.018	0.456
Conservative attitudes toward sexual activities	0.065	0.009	2.432	0.015	0.028	0.017	1.054	0.292	1.091	0.026	1.036–1.149	<0.001
Attitudes toward equal dating relationship	0.121	0.015	4.249	<0.001	0.118	0.027	4.139	<0.001	1.141	0.040	1.056–1.234	<0.001
Positive relationship with school teacher	0.106	0.015	3.763	<0.001	0.094	0.027	3.291	<0.001	1.133	0.041	1.045–1.228	0.003

The factors on the recognition of physical and psychological violence selected by multiple regression analyses

The factors on the recognition of sexual violence selected by binary logistic regression analyses

**Table 3:** Multiple regression and binary logistic regression analyses of the effects of selected factors on the recognition of dating violence sings among girls

Girls (*n* = 1503)	Physical violence				Psychological violence				Sexual violence			
	*β*	SE	*t*	*p* value	*β*	SE	*t*	*p* value	Exp (B)	SE	95 % CI	*p* value
Knowledge of dating violence	0.080	0.011	2.875	0.004	0.122	0.021	4.368	<0.001	1.172	0.034	1.097–1.252	<0.001
Self-esteem	0.002	0.004	0.069	0.945	0.005	0.008	0.177	0.860	0.999	0.014	0.972–1.027	0.943
Conservative attitudes toward sexual activities	0.107	0.010	3.894	<0.001	0.105	0.019	3.804	<0.001	1.172	0.030	1.104–1.244	<0.001
Attitudes toward equal dating relationship	0.118	0.015	4.186	<0.001	0.097	0.028	3.442	<0.001	1.103	0.045	1.009–1.205	0.031
Positive relationship with school teacher	0.003	0.014	0.109	0.913	0.013	0.025	0.444	0.657	1.054	0.042	0.970–1.144	0.212

The factors on the recognition of physical and psychological violence selected by multiple regression analyses

The factors on the recognition of sexual violence selected by binary logistic regression analyses

Table 2 shows that the KDV, CATSA, ATEDR, and PRWST scores were significant predictors for the ability of boys to recognize the signs of physical and sexual violence. Regarding the ability to recognize the sign of psychological violence, the KDV, ATEDR, and PRWST scores were shown to be significant predictors for boys.

Table 3 shows that the KDV, CATSA, and ATEDR scores were significant predictors for the ability of girls to recognize the signs of physical, psychological, and sexual violence. However, PRWST scores were not significant predictors for the ability to recognize the signs of physical, psychological, and sexual dating violence.

## Discussion

This study was performed to examine the recognition of the signs of dating violence by Japanese junior high school students. We determined the association with gender, the KDV score, self-esteem, the CATSA score, and the ATEDR score on their ability to recognize the signs of dating violence.

### Gender differences

When gender differences were investigated, it was revealed that girls had higher KDV, ATEDR, and PRWST scores than boys. Girls knew more about the KDV items than boys. This finding is similar to reports of Western researchers, who have also found that girls tend to display greater knowledge of dating violence when they investigated high school students and college students [19]. These gender differences in the level of knowledge of dating violence may be due to the fact that women are more likely to be the victims of such violence and that they are thus more interested in it compared with boys among the population aged 13–17 years [20].

The ATEDR scores indicated that girls placed more importance on an equal dating relationship than boys. This finding is similar to the report that girls are more supportive of equality in dating relationships among junior high school students [21]. The present study showed that even among junior high school students, there was a significant gender difference of attitudes toward gender equality in dating relationships. In Japan, the Basic Act for a Gender Equal Society was promulgated in 1999 and education was commenced in order to achieve such a society [2]. However, dominance of men over women is still ingrained in Japanese society and the aspirations of women are belittled, especially in rural areas where *conservative attitude* dictate that a woman should be a dutiful wife and devoted mother [22]. Accordingly, *conservative attitude* may have influenced the participating students because this study was performed in rural district in Japan.

Girls had higher scores for the PRWST items than boys. This finding is similar to another report that girls tend to communicate with teachers more frequently in junior high school [11]. The present study showed that there was a gender difference with respect to communicating with school teachers.

Boys had higher self-esteem than girls. The total score for self-esteem was similar to the scores obtained in other studies of junior high school students [11, 16], and it has been previously reported that boys have higher self-esteem than girls [11].

Tomiyasu [23] reported there were statistically significant gender differences that men were more likely to recognize the sign of dating violence compared with women among Japanese high school students and college students between the ages of 16 and 25. Therefore, these results suggest a gender difference by the recognition of signs of dating violence. In this study, there were no gender differences with regard to the tree scores of the students on the RSDV among junior high school students between the ages of 13 and 15. We supposed that it might be the difference in the recognition of signs of dating violence by age.

### Predictors of recognizing of the signs of physical, psychological, and sexual dating violence

First, we found that boys with higher scores on the KDV, CATSA, ATEDR, and PRWST scales tended to have a greater ability to recognize the signs of physical and sexual violence. Second, boys who had higher scores on the KDV, ATEDR, and PRWST scales displayed more ability to recognize the signs of psychological violence. Third, we showed that girls who had higher scores on the KDV, CATSA, and ATEDR scales tended to demonstrate better recognition of the signs of physical/psychological and sexual violence.

### Knowledge of dating violence

The KDV score was a significant predictor of the ability of students (both boys and girls) to recognize the signs of physical/psychological and sexual dating violence. That is, the boys and girls who had higher KDV scores showed greater recognition of the signs of dating violence. This finding is similar to the results of a study performed in Japanese high school students and college students. Tomiyasu et al. [23] reported that scores for recognition of the signs of dating violence were higher after high school students received education to improve knowledge of dating violence than before education. In addition, the majority of college students who displayed appropriate knowledge of dating violence showed a greater ability to recognize the signs of violent behavior such as dating violence [5].

### Self-esteem

Self-esteem was not a significant predictor for recognition of the signs of dating violence. Denny et al. reported that students who attended classes designed to delay sexual activity by focusing on improvement of self-esteem commenced sexual activity with their partners at an older age than students who did not attend such classes in junior or high school [24]. In Japan, it has also been reported that there is an association between the sexual activity of junior or high school students and their level of self-esteem [16], while the effectiveness of a sex education program for enhancing self-esteem has been demonstrated in junior high school students [25]. It is possible that no association was observed between self-esteem and recognition of the signs of dating violence in the present study because we did not examine the relation between self-esteem and experience of sexual dating violence.

### Conservative attitude toward sexual activity

The CATSA score was a significant predictor of the ability of students (both boys and girls) to recognize the signs of physical and sexual dating violence. The Japanese Association for Sex Education [10] reported that individuals with more sexual experience tend to show decreased awareness of risky sexual behavior among junior high school students or high school students. Therefore, individuals with a conservative attitude toward sexual activity may be more likely to be aware of risky sexual behavior, such as sexual violence, and thus have a greater ability to recognize the signs of physical and sexual dating violence. Indeed, Simons et al. [26] suggested that individuals with permissive and relaxed attitudes toward sexual activity may believe that sexual intercourse is acceptable on the first date and that dating violence may be more common among persons with a liberal attitude toward sexual activity among college males.

Girls who had higher CATSA scores showed a greater ability to recognize the signs of psychological dating violence. On the other hand, the CATSA score was not a significant predictor for recognition of the signs of psychological dating violence by boys. This finding is similar to reports of a study, recognition of the signs of psychological dating domestic violence and conservative attitude toward sexual intercourse girls without sexual experience showed higher scores than those experiencing sexual intercourse among Japanese junior high school students [27]. On the other hand, recognition of signs of psychological dating domestic violence was not a significant predictor for boys without sexual experience [27]. Another study showed that experience with sexual intercourse, friends with sexual experiences, and older dating partners were predictors of attitude toward sexual activity among junior high school students [17]. Thus, there were possible difference about predictor factors of attitude toward sexual activity between boys and girls. Therefore, we suggested that there was gender difference with attitude toward sexual activity associated with the ability of recognize the sign of psychological dating violence.

### Attitude toward an equal dating relationship

The ATEDR score was another significant predictor of the ability of students (both boys and girls) to recognize the signs of physical/psychological and sexual dating violence. Boys and girls who placed greater emphasis on an equal dating relationship showed more ability to recognize the signs of dating violence. Earlier researchers have stated that an egalitarian attitude is essential for preventing violence against women, while strongly egalitarian individuals endorse greater equality between men and women in terms of power, rewards, and burdens [28]. These findings indicate that individuals with egalitarian gender roles might see interpersonal relationships as being more equal and might be less likely to support violence against women. Therefore, individuals who emphasize an equal dating relationship may not tolerate dating violence. Interestingly, it was reported that subjects who received education about dating violence along with education regarding prevention tended to show stronger support for an equal romantic relationship than subjects who did not receive this education among junior high school students [18]. In this context, research into the relationship between sex role attitudes and violence has revealed that individuals with an egalitarian sex role attitude tend to spend less time in violent relationships [29].

### Positive relationship with school teachers

The PRWST score was a significant predictor of the ability of boys to recognize the signs of physical/psychological and sexual violence. Thus, boys who had higher PRWST scores showed greater recognition of the signs of dating violence. This finding is similar to results obtained in Western studies. Some studies have also shown that communication with teachers and school-based interventions influence the sexual behavior of adolescents. For instance, a school-based intervention called “Safer Choices” reduced one or more measures of risky sexual behaviors among all youth groups, and was especially effective for boys in high school [30]. In the United States and Canada, 20 reports on adolescent dating violence were published between 2000 and 2010, with 53 risk factors and 6 protective factors being identified [31]. The protective factors were relationship factors, including a positive relationship with the adolescent’s mother and school attachment [31]. It has been reported that school attachment is a predictor of whether or not male-to-female violence will occur among female high school students [32]. This supports the value of a positive relationship with school teachers for boys. Given that improving relations with school teachers is associated with increasing the effectiveness of dating violence prevention programs, it is important to address this issue together with teachers.

The present study demonstrated that the ability of junior high school students to recognize the signs of dating violence was associated with three factors, which were knowledge of dating violence, a conservative attitude toward sexual activity, and their attitude toward an equal dating relationship. In addition, a good relationship with school teachers was associated with the ability of boys to recognize the signs of dating violence. When education programs are designed to prevent dating violence, the present study suggested that four factors should be incorporated to promote recognition of the warning signs of dating violence. These findings suggest that education programs to prevent dating violence should promote knowledge of dating violence with consideration of gender differences and should foster better relations between students and teachers, as well as promoting establishment of an equal dating relationship between boys and girls. Since the rise of dating violence is coupled with adolescents dating at a younger age, it is crucial for educators to develop an understanding of these factors in order to devise effective programs for junior high school students to participate in before they commence sexual activity.

## Limitations

This study was not free from limitations. Given that we could not collect data from other parts of Japan, replication of the findings should be investigated among junior high school students from other regions. Research performed in a rural district might have shown that age, gender, socioeconomic status, and parent–adolescent relations were factors that influence on attitudes toward dating violence [33]. However, such demographic factors were not included in the present study because we could not get permission from the school principals to ask about parental information, such as income. Therefore, it would be important to investigate such demographic information in future studies. Despite these limitations, this study highlighted the importance of addressing the issue of dating violence among junior high school students.

## References

[1] Silverman JG, Raj A, Clements K (2004). Dating violence and associated sexual risk and pregnancy among adolescent girls in the United States. Pediatrics.

[2] Cabinet Office, Gender Equality Bureau. Assessment of intimate partner violence. http://www.gender.go.jp/public/index.html. Accessed 20 March 2013.

[3] Matsuda Y (2008). The fact about perpetrator of dating violence. Nurs Educ..

[4] Nakata K (2007). Do you know about date domestic violence? Date domestic violence and its prevention education. J Midwifery..

[5] Ohnishi M, Nakao R, Shibayama S, Matsuyama Y, Oishi K, Miyahara M (2011). Knowledge, experience, and potential risks of dating violence among Japanese university students: a cross-sectional study. BMC Public Health..

[6] Halpern CT, Oslak SG, Young ML, Martin SL, Kupper LL (2001). Partner violence among adolescents in opposite-sex romantic relationships: findings from the National Longitudinal Study of Adolescent Health. Am J Public Health.

[7] DeMaris A (1990). The dynamics of generational transfer in courtship violence. A biracial exploration. J Marriage Fam..

[8] Lavoie F, Hebert M, Tremblay R, Vitaro F, Vezina L, McDuff P (2002). History of family dysfunction and perpetration of dating violence by adolescent boys: a longitudinal study. J Adolesc Health.

[9] WHO. Prevention of Intimate partner violence and sexual violence. http://www.who.int/violence_injury_prevention/violence/activities/intimate/en/. Accessed 10 Jan 2012.

[10] Japanese Association for Sex Education (2013). National youth white paper of the youth: survey of Japanese young sexual behavior 2011.

[11] Hara K, Nagamatsu M, Nakagawa A, Saito H (2012). How the frequency of conversation between junior high school students and their parents/teachers is related to the students’ knowledge, awareness, and behavior regarding dating and sexually transmitted diseases. Adolescentology..

[12] Theriot MT (2008). Conceptual and methodological considerations for assessment and prevention of adolescent dating violence and stalking at school. Natl Assoc Soc Work.

[13] Yamaguchi N (2005). A workbook for preventing dating violence.

[14] Rosenberg M (1965). Society and adolescent self-image.

[15] Hoshino A (1970). Psychology of emotion and education. Child Psychol..

[16] Kawabata T, Ishikawa T, Katsuno S, Nishioka N, Nozu Y, Shimai S (2007). Sexual behavior and related factors among junior and senior high school students: focusing on psychosocial variables including self-esteem. Jpn J Sch Health..

[17] Nagamatsu M, Yamawaki N, Sato T, Nakagawa A, Saito H (2013). Factors influencing attitudes toward sexual activity among early adolescents in Japan. J Early Adolesc.

[18] Nagamatsu M, Hara K, Nakagawa A, Nakano R (2012). Efficacy of the program for the prevention of risky sexual behavior: combined education on cultivating mutual respect between both sexes and preventing sexually transmitted diseases. Adolescentology.

[19] Cornelius TL, Resseguie N (2007). Primary and secondary prevention programs for dating violence: a review of the literature. Aggress Viol Behav..

[20] Swain CR, Ackerman LK, Adckerman MA (2006). The influence of individual characteristics and contraceptive beliefs on parent-teen sexual communications: a structural model. J Adolesc Health.

[21] Jaffe PG, Sudermann M, Reitzel D, Killip SM (1992). An evaluation of a secondary school primary prevention program on violence in intimate relationships. Violence Vict.

[22] Saotome T, Hiraiwa T (2011). Current status and problems of the children. Problems of puberty: until education from its present situation.

[23] Tomiyasu T, Suzui M (2014). A study of the usefulness of a date violence prevention education for young unmarried people in Japan. Jpn J Maternak Health..

[24] Denny G, Young M (2006). An evaluation of an abstinence-only education curriculum: an 18-month follow-up. J Sch Health.

[25] Tomioka M (2008). Effects of sexual education for junior high school students accompanied by life-skill training on knowledge, attitude and behavior. Adolescentology..

[26] Simons LG, Burt CH, Simons RL (2008). A test of explanations for the effect of harsh parenting on the perpetration of dating violence and sexual coercion among college males. Violence Vict.

[27] Nagamatsu M, Hara K (2015). Sexual intercourse experience in early adolescence and how it relates to dating domestic violence as an influential factor. Adolesecentogy..

[28] Brighouse H, Wright EO (2008). Strong gender egalitarianism. Polit Soc..

[29] Flynn CP (1990). Sex roles and women’s response to courtship violence. J Fam Violence..

[30] Kirby D, Baumle E, Coyle KK, Basen-Engquist K, Parcel GS, Harrist R (2004). The “Safer Choices” intervention, its impact on the sexual behaviors of different subgroups of high school students. J Adolesc Health.

[31] Vagi KJ, Rothman EF, Latzman NE, Tharp AT, Hall DM, Breiding MJ (2013). Beyond correlates: a review of risk and protective factors for adolescent dating violence perpetration. J Youth Adolesc.

[32] Cleveland HH, Herrera VM, Stuewig J (2003). Abusive males and abused female in adolescent relationships: risk factor similarity and dissimilarity and the role of relationship seriousness. J Fam Violence..

[33] Jones SR, Gardner SP (2002). Variables related to attitudes toward domestic violence and use of reasoning, verbal aggression, and violent conflict tactics in high school students. J Fam Consum Sci Educ.

